# The Effect of Using Virtual Reality on Pain, Fear, and Anxiety in School-Age Children During Venipuncture: A Randomized Controlled Study †

**DOI:** 10.3390/healthcare14172770

**Published:** 2026-09-01

**Authors:** Hamiyet Kızıl, Esra Karaca, Gözde Aksucu, İlayda Azap

**Affiliations:** 1 Hamidiye Faculty of Nursing, University of Health Sciences, Istanbul 54600, Türkiye; 2 Department of Nursing, Faculty of Health Sciences, Istanbul Beykent University, Istanbul 34398, Türkiye; esraciftci@beykent.edu.tr; 3 Department of Child Development, Faculty of Health Sciences, Istanbul Beykent University, Istanbul 34398, Türkiye; gozdeaksucu@beykent.edu.tr; 4 Medicana Ataköy Hospital, Istanbul 34158, Türkiye; ilaydaltintop@gmail.com

**Keywords:** virtual reality, venipuncture, pain, fear, anxiety, school-age children

## Abstract

**Introduction and Aim:** Pain, fear, and anxiety are common during invasive procedures in children. Virtual reality (VR), an attention-distraction method based on cognitive and behavioral techniques, has been increasingly used to reduce these responses. This randomized controlled trial investigated the effect of VR glasses on pain, fear, and anxiety in school-age children undergoing venipuncture (ClinicalTrials.gov ID: NCT06727149). **Method:** This study was conducted between September 2022 and May 2023 in the pediatric inpatient unit of a private hospital in western Turkey. Sixty children aged 6–12 years were randomly assigned to either the VR group (n = 30) or the control group (n = 30). Data were collected using the Child Information Form, Wong–Baker Faces Pain Rating Scale, Children’s Fear Scale, and State-Trait Anxiety Inventory for Children. Statistical analyses were performed using SPSS version 25. **Results:** The groups were comparable in terms of age and sex (*p* > 0.05). Following venipuncture, the VR group had significantly lower mean pain scores than the control group (0.68 ± 0.51 vs. 1.06 ± 0.62, *t* = 2.559, *p* = 0.013). The mean fear score was also significantly lower in the VR group (0.83 ± 0.50 vs. 1.12 ± 0.53, *t* = 2.157, *p* = 0.035). Similarly, anxiety scores were significantly lower in the VR group than in the control group (49.66 ± 7.29 vs. 52.86 ± 4.65, *t* = 2.025, *p* = 0.048). **Conclusions:** VR glasses effectively reduced venipuncture-related pain, fear, and anxiety in school-age children. These preliminary findings support VR as a promising non-pharmacological adjunct for reducing procedural pain, fear, and anxiety; given the single-center design and modest sample size, confirmation in larger, multicenter trials involving more diverse pediatric populations are needed before routine incorporation into pediatric care can be recommended.

## 1. Introduction

Some diagnostic procedures and treatment practices applied to children may cause the child to experience pain, fear and anxiety. Intravenous procedures such as venipuncture, IV cannula placement and injection are the most common invasive procedures that children are exposed to [1,2]. Most children are hesitant to undergo needle-based procedures such as vaccinations and blood collection. In most cases, alternatives to invasive procedures are costly in terms of expense, time or biological side effects. While parents can choose these alternative methods to relieve their children’s pain, such relief may not always be possible. The pain and fear experienced during the procedure can have severe effects that last beyond the duration of the procedure. Studies show that the undesirable consequences of such procedures may cause needle phobia that persists into adulthood in up to 10% of the world’s population. Such patients have been shown to exhibit extreme negative reactions to needles, such as vasovagal reactions and changes in the person’s heart rate and stress hormone levels. Mortality rates may even increase as a result of avoiding necessary medical care [3,4,5,6].

Although VR-based distraction has been investigated in the pediatric procedural-pain literature for over two decades, findings across studies remain inconsistent with respect to fear and anxiety outcomes, and comparatively few randomized trials have examined all three outcomes (pain, fear, and anxiety) concurrently, with parent-, child-, and observer-rated agreement, in school-age children undergoing venipuncture in an inpatient setting. This gap in the literature—the limited concurrent assessment of pain, fear, and anxiety using multi-rater, validated instruments in this specific age group and clinical contextprovides the rationale and scientific justification for the present study.

There are many approaches to reduce pain, fear and anxiety that may occur in children during invasive procedures. The most commonly used of these approaches the distraction technique, which is designed using cognitive and behavioral principles [6,7]. There are many methods used to distract attention. Some of these are; watching cartoons, blowing balloons, creating balloons by blowing bubbles, redirection technique, parental coaching, placebo, hypnosis, using virtual reality (VR) glasses, listening to music, kaleidoscope and using distraction cards [8,9,10].

The evidence supporting these distraction approaches, including VR, is not uniformly positive: while several randomized trials and meta-analyses report significant reductions in procedural pain, effects on fear and anxiety are less consistent, and some studies report only modest or non-significant differences between VR and control conditions. A critical appraisal of these mixed findings, rather than reliance on studies with positive results alone, is necessary to justify further investigation of VR in this population.

In order to temporarily distance the individual from real life, the individual/patient is given the feeling of visiting another world in three dimensions by using VR glasses, which are a method of watching images taken from a computer with a headset attached to the patient. Appealing to more than one sense, VR has an advantage over other distraction techniques because it offers a convincing, immersive and attractive world and attracts the attention of children more. Beyond simple distraction, VR is thought to modulate the experience of pain through several neuropsychological mechanisms: it competes for limited attentional resources, so that fewer cognitive resources remain available to process nociceptive input (attentional resource theory); it alters the perceived intensity and unpleasantness of noxious stimuli by engaging higher-order cortical and subcortical circuits; and, consistent with gate control theory, it may promote the closing of the spinal “gate” for pain transmission by activating competing non-nociceptive sensory and cognitive pathways. These mechanisms provide a stronger theoretical rationale for VR’s effects not only on pain but also on fear and anxiety, which involve overlapping attentional and emotional-regulation processes. When deciding which non-pharmacological method to choose to reduce pain, fear and anxiety during invasive procedures, it is important to keep up with the information and technology age, as well as considering the child’s age and cognitive competence, interests, culture, ability to cope with the situation and the type of pain. Although VR has been clinically investigated as a distraction technique for more than two decades, recent developments concern primarily more advanced, portable, and immersive systems, which have broadened its accessibility and feasibility for routine pediatric care [10,11,12].

Virtual reality (VR) can be classified according to the level of immersion experienced by the user. This varies between non-immersion (desktop) systems, semi-immersion projection systems, and full immersion VR systems. Full immersion VR is a newer form of distraction and has only really gained traction as a viable form of pain, fear, and anxiety management in the last decade. This is largely due to more recent developments in the field, allowing for increased portability, ease of use, and accessibility. This technology includes 3D-enabled glasses, headsets, and sensory input devices or even a head-mounted device with body-tracking sensors, providing a multisensory experience. VR therapy also involves multiple neural cortical and subcortical circuits that enhance healing and learning for patients. It can be used in pediatrics, particularly for pain management, as it can be easily adapted to painful procedures [1,2,8,9,10]. In light of this information, it is thought that the use of VR glasses during venipuncture in children may offer the opportunity to reduce pain, fear, and anxiety.

The present study focused on children aged 6–12 years because, by this stage of cognitive development, most children can understand simple instructions, use symbolic and abstract reasoning, and reliably differentiate and self-report their own pain, fear, and anxiety using structured scales such as the Wong–Baker Faces Pain Rating Scale, the Children’s Fear Scale, and the State-Trait Anxiety Inventory for Children. This age range is also old enough to operate VR headsets safely and cooperatively, while remaining within the age band for which the study instruments have been validated, supporting the reliability of self-reported outcomes in this population.

Beyond its role as a distraction tool for procedural pain, VR is increasingly recognized as capable of influencing a broader range of psychological states relevant to pediatric procedural distress, including the sense of flow and engagement it can induce, its effects on anxiety, enjoyment, perceived control, and emotional regulation, and, in some contexts, its potential negative effects such as cybersickness or heightened distress in anxious children. These psychological mechanisms, rather than distraction alone, provide additional theoretical justification for examining fear and anxiety not only pain as primary outcomes in the present study [13,14,15,16,17,18,19]. Accordingly, this study was conducted to determine the effect of VR glasses on pain, fear, and anxiety levels in school-age children aged 6–12 years undergoing venipuncture.

## 2. Materials and Methods

This study aimed to determine the effect of virtual reality (VR) glasses provided to children during the venipuncture procedure on pain, fear, and anxiety. This study aimed to determine the effect of virtual reality (VR) glasses provided to children during the venipuncture procedure on pain, fear, and anxiety.

### 2.1. Research Hypotheses

Pain, fear and anxiety are studied separately in this study; therefore, three hypotheses are proposed. In line with previous research, it is hypothesized that VR glasses used during the venipuncture procedure have positive effects on children’s pain (H_1_), fear (H_2_) and anxiety (H_3_).

### 2.2. Study Design

This randomized controlled clinical trial (RCT) was conducted between September 2022 and May 2023 with two parallel groups in the pediatric inpatient service of a private hospital in western Türkiye.

During the planning phase of the study, a number was obtained from the official institution called ClinicalTrials, which is managed by the National Institutes of Health (NIH), National Libraries of Medicine (NLM), Office of Management and Budget (OBM), Department of Health and Human Services (DHHS), which evaluates and approves experimental research internationally (ClinicalTrials.gov ID: NCT06727149-12/06/2024).

The study was registered on ClinicalTrials.gov after participant recruitment began. This is a retrospective registration, not a prospective one, which represents a limitation of the study. The registration was completed as soon as this requirement was identified, and no changes were made to the study protocol, outcomes, or analysis plan after data collection had begun.

### 2.3. Setting and Participants

In order to determine the number of patients constituting the research sample, a similar study in the relevant literature [19] was taken as an example and the power analysis G*Power 3.1 software was used. In this study, in order to reach a power level of 95% at a 0.5 effect size and 5% error level, the sample size was calculated as 56, with 28 participants in each group. Considering the high power of the test and the losses in the study, a total of 60 people were reached, 30 in each group. The study sample consisted of children aged 6–12 years with stable hemodynamics who were undergoing venipuncture, had sufficient cognitive and communication abilities to rate their pain, fear, and anxiety levels, and were willing to participate. This group was selected because children of that age are the most cooperative. All participating children gave verbal consent to participate, and their families provided written consent. Children with unstable conditions and vital signs, intubated, had visual problems or did not want to participate in the study were excluded.

### 2.4. Randomization

**Figure 1:**
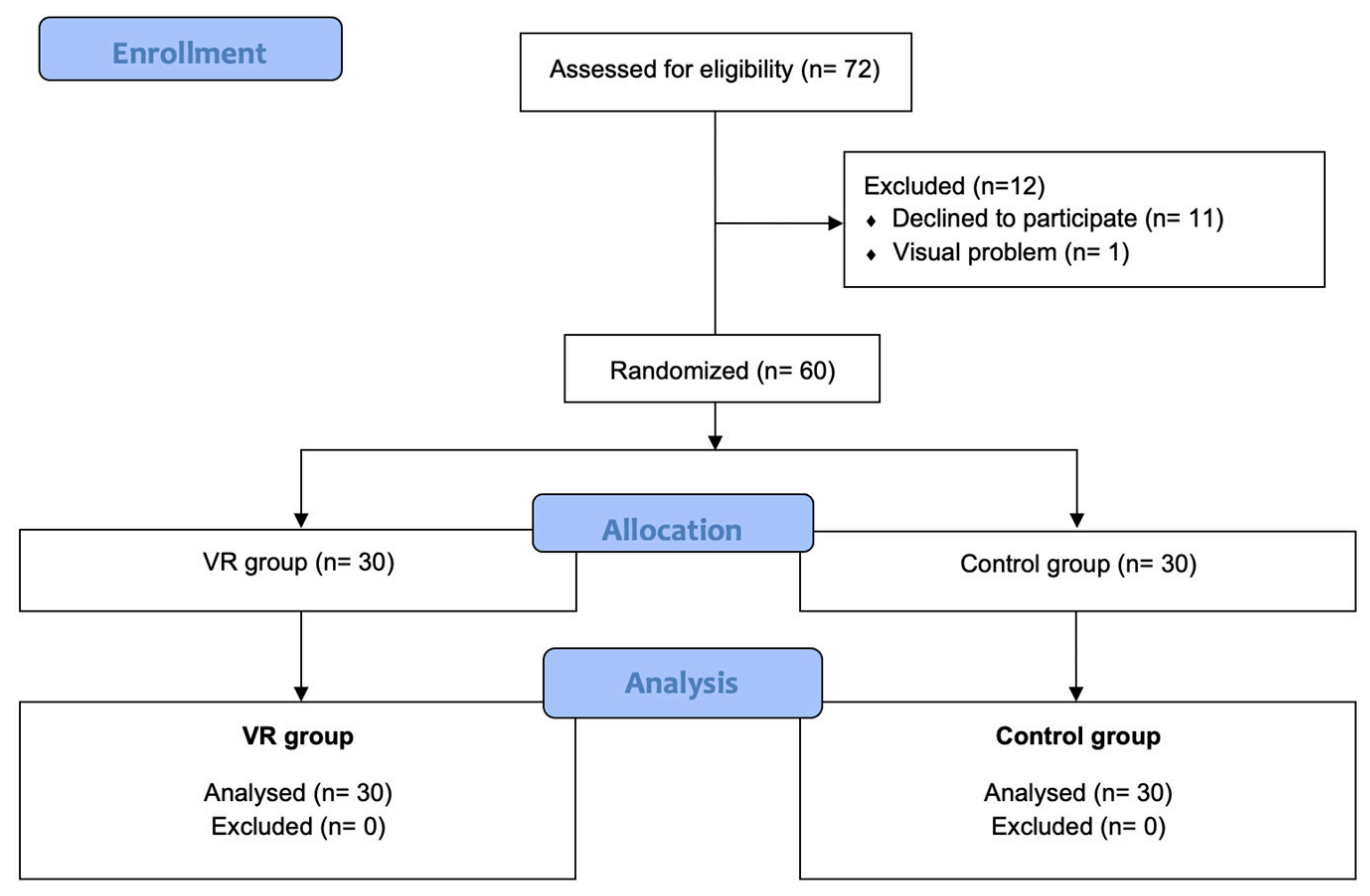
CONSORT flow diagram.

Randomization was performed using a computer-based random number generator (StatTrek) with a simple randomization method. The randomization sequence was generated by a researcher who was not involved in data collection, the venipuncture procedure, or outcome assessment. Based on the computer-generated randomization sequence, 60 identical opaque cards, 30 marked “VR” and 30 marked “control,” were prepared and placed in a closed box to maintain allocation concealment. At enrollment, each participant drew one card from the box and was assigned to the corresponding group. Thus, 60 children were included in the study, with 30 participants in the VR group and 30 in the control group. The CONSORT flow diagram is presented in Figure 1.

Because the VR intervention was visible during venipuncture, blinding of the children, parents, the researcher performing the venipuncture (H.K.), and the researchers involved in data collection (İ.A. and G.A.) to group allocation was not feasible. Therefore, the study was conducted as an open-label randomized controlled trial. No independent observer blinded to group allocation was used. However, to minimize potential assessment bias, child-, parent-, and researcher-reported ratings were recorded independently, without access to one another’s scores at the time of assessment. The researcher who generated the randomization sequence had no role in the venipuncture procedure, data collection, or outcome assessment.

Although participants were aware of the intervention received during venipuncture, they were not informed of the specific study hypotheses or the expected differences between the groups. The same standardized data collection and assessment procedures were applied to both groups to ensure consistency and minimize potential assessment bias.

### 2.5. Measurements

The research’s outcomes were pain levels assessed with the Wong–Baker Faces Pain Rating Scale (WBS), fear levels assessed with the Children’s Fear Scale (CFS), anxiety levels assessed with the State-Trait Anxiety Inventory for Children (STAI-CH). WBS and CFS were scored separately by the researcher, the child and the parent, and STAI-CH was scored by the child alone within five minutes before and immediately after the procedure. All ratings were done blindly to ensure that the observers did not influence each other’s scores.

**Child Information Form:** The researchers created the Child Information Form and included nine questions about the children’s age, sex, parents’ age, education level, economic level, and type of family.

**Wong–Baker Faces Pain Rating Scale:** The WBS consists of six cartoon faces with expressions ranging from smiling (no pain, 0 points) to crying (worst pain, 10 points) and was developed for the assessment of pain intensity in children from 3 years old [20]. Turkish validity and reliability studies of the WBS have been conducted, and it is frequently used for Turkish children [9,10,12]. The children’s pain scores before and after venipuncture were rated by the study team member who specializes in pediatric nursing, the children, and the parent.

**Children’s Fear Scale:** The CFS is a single-item scale used to assess pain-related fear in children. This scale comprises a series of facial expressions ranging from no fear (0 points) to very fearful (4 points) [11,12]. It can be used with children 5–12 to assess fear before and during a procedure [11]. Turkish validity and reliability studies of the CFS have been conducted, and it is frequently used for Turkish children [12,21]. The children’s fear scores before and after venipuncture were rated by the study team member who specializes in pediatric nursing, the children, and the parent. Turkish validity and reliability studies of the CFS have been conducted [22].

**State-Trait Anxiety Inventory for Children:** The STAI-CH is the instrument for measuring anxiety in children. It distinguishes between a general proneness to anxious behavior rooted in the personality and anxiety as a fleeting emotional state. This scale is designed for upper elementary or junior high school-aged children and consists of two twenty-item scales [23].

### 2.6. Procedure

One researcher (İ.A.) provided information about the study to the children and their parents in the patient rooms. Children who met the inclusion criteria were enrolled in the study, and written informed consent was obtained from the parents and assent from the children. Participants were then randomly assigned to the VR or control group, and the Child Information Form was completed.

Before the venipuncture procedure, the children were informed that intravenous access would be established and were given standardized instructions on how to complete the assessment scales. The children rated their pain and fear using the Wong–Baker Faces Pain Rating Scale (WBS) and the Children’s Fear Scale (CFS), respectively, and their anxiety using the State-Trait Anxiety Inventory for Children (STAI-CH). Parents independently rated their child’s fear using the CFS. The children and parents completed their ratings without seeing each other’s scores. Researcher İ.A. also independently rated the child’s pain and fear using the WBS and CFS without access to the child’s or parent’s ratings. Although the researchers were aware of group allocation, the ratings were recorded independently without access to one another’s scores to minimize potential assessment bias.

All children were allowed to have a parent present during the venipuncture procedure. To ensure procedural consistency, venipuncture was performed by the same researcher (H.K.) for all participants. H.K. performed the venipuncture procedure but was not involved in collecting or scoring the outcome measures. Before the procedure, the materials were introduced to the child, and the child was asked which arm they preferred for venipuncture. The procedure was performed after obtaining the child’s cooperation. If the first attempt was unsuccessful, permission was sought from the child before attempting venipuncture on the other arm. If the child did not wish to undergo the procedure immediately, the procedure was postponed until the child indicated readiness.

Children assigned to the VR group were informed about the VR headset by researcher İ.A. and were told that they could remove it at any time if they experienced discomfort during the procedure. Immediately before venipuncture, children wore an Oculus Quest 2 VR headset and passively viewed an age-appropriate three-dimensional distraction video. The standardized 3D distraction video was approximately 10 min in duration, which was sufficient to cover the expected duration of the venipuncture procedure. The VR intervention was non-interactive; children were not required to control, navigate, or otherwise actively interact with the virtual environment. To standardize the intervention, all children in the VR group viewed the same video content in the same sequence. VR exposure began immediately before venipuncture and continued until the procedure was completed.

The three-dimensional distraction video was developed by the researchers using computer-based video-editing software. To ensure intervention consistency and reproducibility, the same VR headset, video content, presentation sequence, and viewing procedure were used for all participants in the VR group.

In the control group, no distraction intervention was applied during venipuncture. Children received routine venipuncture care only, and a parent was allowed to remain present during the procedure, consistent with the VR group.

**Figure 2:**
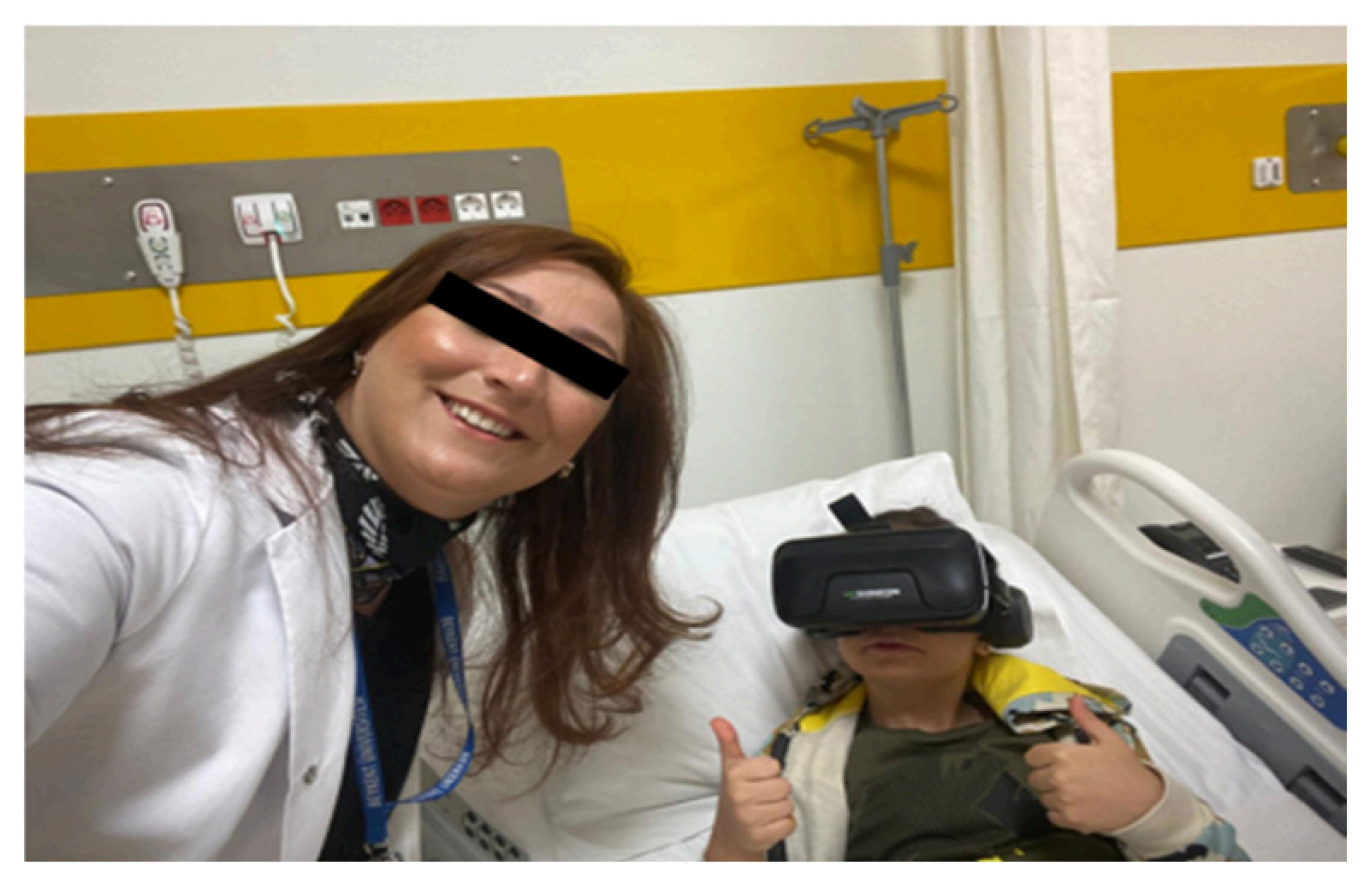
A school-age child using virtual reality-based distraction during venipuncture.

Immediately after venipuncture, pain and fear were assessed using the WBS and CFS, respectively. The child, parent, and researcher (İ.A.) recorded their respective ratings independently and without access to one another’s scores. The child also completed the STAI-CH to assess post-procedural anxiety. Although the assessors were aware of group allocation because of the visible nature of the VR intervention, ratings were recorded independently to minimize potential assessment bias. Figure 2 presents a photograph illustrating the use of the VR headset during the venipuncture procedure. Written parental consent and the child’s assent were obtained for publication of the photograph.

### 2.7. Data Analysis

The data obtained in the study were analyzed using SPSS (Statistical Package for the Social Sciences) for Windows, version 22.0. Descriptive statistics, including number, percentage, mean, and standard deviation, were used to summarize the data. Differences in categorical variables between the independent groups were analyzed using the chi-square test or Fisher’s exact test, as appropriate. Independent-samples *t*-tests were used to compare continuous variables between the VR and control groups, while paired-samples *t*-tests were used to compare pre- and post-procedure measurements within groups. Relationships between the dependent variables were examined using Pearson correlation and regression analyses. Statistical significance was set at *p* < 0.05.

Prior to hypothesis testing, the normality of the continuous outcome variables (WBS, CFS, and STAI-CH scores) was assessed using the Shapiro–Wilk test. Based on the normality assessment and examination of the distributions, the continuous outcome variables were considered approximately normally distributed. Given the equal sample sizes in the two groups and the approximate normality of the distributions, parametric tests were considered appropriate. Although the WBS and CFS are ordinal in construction, they were analyzed as approximately interval-level measures using independent- and paired-samples *t*-tests. The absence of complementary non-parametric sensitivity analyses, such as the Mann–Whitney U and Wilcoxon signed-rank tests, was acknowledged as a limitation of the study.

The internal consistency of the multi-item State-Trait Anxiety Inventory for Children (STAI-CH) was high in the present study (Cronbach’s α = 0.885). Because the WBS and CFS are single-item instruments, Cronbach’s alpha was not considered an appropriate measure of internal consistency for these scales. Instead, agreement among the child-, parent-, and researcher-reported ratings was assessed using a multi-rater kappa statistic. Agreement among the three rating sources was high for pain (κ = 0.789, *p* < 0.001) and fear (κ = 0.816, *p* < 0.001). Because researcher İ.A. was aware of group allocation, these coefficients represent agreement among the three rating sources rather than agreement among blinded assessors.

Effect sizes and 95% confidence intervals (CIs) were also calculated to complement the significance tests. The between-group difference in post-procedure fear corresponded to a medium effect size (Hedges’ g = −0.56, 95% CI: −1.08 to −0.05; mean difference = −0.29, 95% CI: −0.56 to −0.02). The within-group pre–post reduction in fear in the VR group corresponded to a large effect (Cohen’s dz = 0.80, 95% CI: 0.39 to 1.21), whereas the reduction in the control group was small and non-significant (Cohen’s dz = 0.23, 95% CI: −0.13 to 0.59).

### 2.8. Ethical Considerations

The study was conducted in accordance with the principles of the Declaration of Helsinki. Ethical approval was obtained from the Beykent University Social and Human Sciences Ethics Committee (Decision No: 251, Date: 2 August 2022), and institutional permission was obtained from the hospital where the data were collected.

As the participants were children under 18 years of age, written informed consent was obtained from their parents or legal guardians before enrollment. In addition, verbal assent appropriate to the children’s age and developmental level was obtained from each child. The children and their parents or legal guardians were provided with detailed information regarding the purpose, scope, duration, and procedures of the study, the expectations of participants, and the intended use of the collected data through an Informed Consent Form prepared in accordance with the Declaration of Helsinki. They were given sufficient opportunity to ask questions and receive detailed explanations before deciding whether to participate. Separate written consent was also obtained from the parents or legal guardians for the use of participants’ images in scientific publications.

Prior to data collection, written permission to use the study instruments was obtained via e-mail from the original developers of the measurement scales.

Participants and their parents or legal guardians were assured that all personal information would remain confidential, would be accessible only to the research team, would not be disclosed to third parties, and would be used solely for the purposes of this research. The principles of confidentiality and privacy were strictly maintained throughout the study.

Upon completion of the study, children in the control group were also offered the opportunity to experience the virtual reality intervention to ensure fairness and equal access to the educational intervention in accordance with ethical principles.

## 3. Results

The descriptive characteristics of the children and their parents in the intervention and control groups are summarized in Table 1. Children’s sex and age group were homogeneous (*p* > 0.05). The type of family and the parent’s age, level of education, and economic level were also homogeneous (*p* > 0.05) (Table 1).

Mean pain scores before and after the venipuncture procedure are given in Table 2. Before the procedure, there were no statistically significant differences in mean pain scores among the VR and control groups (*p* > 0.05). After the procedure, children in the VR group had a lower mean pain score than the control group (VR = 0.68 ± 0.51, Control = 1.06 ± 0.62, t = 2.559, *p* = 0.013). The decrease between pretest and post-test pain scores was significant in the VR group (t = 5.193, *p* = 0.000) (Table 2). There was high agreement between the pain scores the children, parents, and researcher gave in the VR and control groups both before and after the venipuncture procedure (Kappa (κ) = 0.789, *p* = 0.000). The between-group difference in post-procedure pain corresponded to a medium effect size (Hedges’ g = −0.66, 95% CI: −1.19 to −0.15; mean difference = −0.38, 95% CI: −0.67 to −0.09), and the within-group pre–post reduction in the VR group corresponded to a large effect (Cohen’s dz = 0.95, 95% CI: 0.52 to 1.38), compared with a small, non-significant reduction in the control group (dz = 0.36, 95% CI: −0.01 to 0.73).

Mean fear scores before and after the venipuncture procedure are given in Table 3. Before the procedure, there were no statistically significant differences in mean fear scores among the VR and control groups (*p* > 0.05). After the procedure, children in the VR group had a lower mean fear score than the control group (VR = 0.83 ± 0.50, Control = 1.12 ± 0.53, t = 2.157, *p* = 0.035). The decrease between pretest and post-test fear scores was significant in the VR group (t = 4.397, *p* = 0.000) (Table 3). There was high agreement between the fear scores the children, parents, and researcher gave in the VR and control groups both before and after the venipuncture procedure (Kappa (κ) = 0.816, *p* = 0.000).

Mean anxiety scores before and after the venipuncture procedure are presented in Table 4. Before the procedure, there was no statistically significant difference in mean anxiety scores between the VR and control groups (*p* > 0.05). After the procedure, the mean anxiety score was significantly lower in the VR group than in the control group (VR = 49.66 ± 7.29; control = 52.86 ± 4.65; t = 2.025, *p* = 0.048). Within the VR group, anxiety scores decreased significantly from pre- to post-procedure (t = 3.257, *p* = 0.003), whereas the reduction in the control group was not statistically significant (Table 4).

The between-group difference in post-procedure anxiety corresponded to a medium effect size (Hedges’ g = −0.52, 95% CI: −1.04 to −0.01; mean difference = −3.20, 95% CI: −6.36 to −0.04). However, this finding should be interpreted cautiously because the corresponding *p* value (*p* = 0.048) was close to the conventional threshold for statistical significance. The pre–post reduction in anxiety in the VR group showed a medium effect size (Cohen’s dz = 0.59, 95% CI: 0.21 to 0.98), whereas the reduction in the control group was smaller and not statistically significant (dz = 0.34, 95% CI: −0.03 to 0.70).

## 4. Discussion

Venipuncture is one of the most frequently performed invasive procedures in hospitalized pediatric patients. Pediatric patients, particularly children aged 6–12 years, often experience high levels of pain, fear, and anxiety during this procedure. Poorly managed pain can contribute to fear and anxiety, increase the time and resources required to perform venipuncture, and reduce children’s and parents’ satisfaction with the procedure. Therefore, clinical guidelines emphasize the importance of effectively managing pain, fear, and anxiety in pediatric patients [5,6,7,8,9,10]. Distraction is one of the most frequently used non-pharmacological interventions in clinical settings to manage pain, fear, and anxiety in pediatric patients undergoing needle-related procedures such as venipuncture. Compared with other non-pharmacological interventions, such as hypnosis and cognitive behavioral therapy, distraction is relatively easy to implement and does not require specialized training [1,2,3,4,8]. The use of virtual reality as a distraction intervention during venipuncture may provide an opportunity to reduce pain, fear, and anxiety in school-age children. Based on the present findings, the use of VR glasses during venipuncture was associated with lower levels of pain, fear, and anxiety in school-age children.

Rather than relying solely on consistency with previous studies, it is important to consider why VR may have been effective in this specific population. As outlined in the Introduction, VR is thought to act through competition for limited attentional resources, altered central processing of nociceptive input, and gate-control-related mechanisms, while also engaging psychological processes such as engagement, perceived control, and emotional regulation. These overlapping mechanisms may plausibly explain why the intervention was associated with reductions not only in pain but also in fear and anxiety in the present sample [13,14,15,16,17,18].

After the venipuncture procedure, the mean pain score of children in the VR group was significantly lower than that of children in the control group (VR = 0.68 ± 0.51, control = 1.06 ± 0.62, t = 2.559, *p* = 0.013). The pre–post reduction in pain scores was statistically significant in the VR group (*p* < 0.001). Previous studies have reported that VR is effective in reducing pain associated with venipuncture in children, supporting the findings of the present study [5,6,7,8,9,10]. Anwr et al. (2024) showed that VR reduced pain during phlebotomy in children [24]. Gerçeker et al. (2024) found that both VR and stress-ball interventions were effective in reducing pain during phlebotomy and reported favorable effects of VR on distraction and negative emotional behaviors [25]. Kanad et al. (2024) found that children in the VR group undergoing blood collection in a pediatric hematology–oncology outpatient clinic had lower post-procedural pain scores and fewer negative emotional responses than those in the control group [19]. Lee et al. (2024) demonstrated that immersive VR was more effective in reducing pain and distress than video-based tablet education among pediatric patients aged 4–8 years undergoing venipuncture [26]. Yılmaz and Canpulat (2023) reported lower pain levels among children aged 7–12 years who used VR during phlebotomy compared with controls [27]. Wong and Choi (2023) showed that VR was more effective than standard care in reducing pain among pediatric patients undergoing venipuncture [28]. Ryu et al. (2022) reported that VR during venipuncture significantly reduced pain and was associated with fewer repeated attempts and lower procedural difficulty [29]. Barad et al. (2020) suggested that VR and cold vibration were similarly effective in relieving phlebotomy-related pain in children aged 5–10 years [30]. İnangil et al. (2020) reported that cartoon-based distraction delivered through a VR device reduced pain during phlebotomy in school-age children [31]. Overall, these findings are consistent with the pain-related results of the present study [24,25,26,27,28,29,30,31]. Accordingly, Hypothesis 1 was supported.

From a clinical perspective, the medium effect size observed for post-procedure pain (Hedges’ g = −0.66) suggests that the reduction associated with VR was not limited to statistical significance alone. However, the relatively modest absolute between-group difference of 0.38 points on the WBS indicates that the clinical relevance of the observed effect should be interpreted not solely on the basis of statistical significance, but also in conjunction with the effect size and the clinical importance of the observed difference.

After the venipuncture procedure, the mean fear score of children in the VR group was significantly lower than that of children in the control group (VR = 0.83 ± 0.50, control = 1.12 ± 0.53, t = 2.157, *p* = 0.035). The pre–post reduction in fear scores was statistically significant in the VR group (*p* < 0.001). Previous studies have reported beneficial effects of VR on fear associated with venipuncture in children, supporting the findings of the present study [1,19,25]. Gerçeker et al. (2024) found that both VR and stress-ball interventions were effective in reducing fear during phlebotomy [25]. Kanad et al. (2024) reported lower post-procedural fear scores among children in the VR group during blood collection in a pediatric hematology–oncology outpatient clinic compared with the control group [19]. EW and Mohamed (2023) reported that Flippit cards and VR reduced pain and fear and increased satisfaction during phlebotomy in children [32]. In a systematic review and meta-analysis examining VR for the management of pain and anxiety in children undergoing venipuncture, Niaz et al. (2023) reported evidence suggesting that VR may be more effective than traditional non-pharmacological approaches for reducing procedural distress [33]. Overall, findings from the literature are consistent with the fear-related results of the present study [32,33,34]. Accordingly, Hypothesis 2 was supported.

Similar to the pain findings, the medium effect size observed for fear (Hedges’ g = −0.56) suggests a potentially clinically relevant, although moderate, benefit of VR. However, given the modest absolute between-group difference, this finding should be regarded as supportive rather than conclusive evidence of a clinically important effect until replicated in larger and more diverse samples.

After the venipuncture procedure, the mean anxiety score of children in the VR group was lower than that of children in the control group (VR = 49.66 ± 7.29, control = 52.86 ± 4.65, t = 2.025, *p* = 0.048). Within the VR group, anxiety scores decreased significantly from pre- to post-procedure (t = 3.257, *p* = 0.003), whereas the reduction observed in the control group was not statistically significant. Previous studies have reported beneficial effects of VR on anxiety during venipuncture in children, generally supporting the findings of the present study [24,25,27,28]. Anwr et al. (2024) reported that VR reduced anxiety during phlebotomy in children [24]. Gerçeker et al. (2024) found that VR and stress-ball interventions were effective in reducing anxiety during phlebotomy [25]. Gold et al. (2024) reported lower anxiety levels among patients receiving VR during venipuncture compared with controls [35]. Wong and Choi (2023) demonstrated that VR was more effective than standard care in reducing anxiety among pediatric patients undergoing venipuncture [28]. Yılmaz and Canpulat (2023) reported significantly lower anxiety levels among children who used VR during phlebotomy than among those in the control group [27]. Ryu et al. (2022) concluded that VR during venipuncture was effective in reducing anxiety and was associated with high parental satisfaction [29]. Gold et al. (2021) reported that children using VR during peripheral intravenous catheter placement experienced significantly less anxiety and pain than those receiving standard care [36]. İnangil et al. (2020) reported that watching cartoons through VR glasses reduced anxiety during phlebotomy in school-age children [31]. Overall, these findings are consistent with the anxiety-related results of the present study [29,30,31,32,33,34,35,36]. Accordingly, Hypothesis 3 was supported; however, the strength of this conclusion should be considered in light of the borderline between-group statistical significance.

Specifically, the between-group difference in post-procedure anxiety was close to the conventional threshold for statistical significance (*p* = 0.048) and corresponded to a medium effect size with a 95% confidence interval approaching zero (Hedges’ g = −0.52, 95% CI: −1.04 to −0.01). Therefore, the evidence for an anxiety-reducing effect of VR in the present sample should be interpreted more cautiously than the findings for pain and fear. Rather than being regarded as conclusive evidence, this result should be considered preliminary and warrants confirmation in adequately powered, larger, multicenter trials.

Taken together, the findings suggest that VR may provide a feasible non-pharmacological adjunct for managing venipuncture-related procedural distress in school-age children. Its potential benefit may extend beyond simple distraction by engaging attentional, sensory, and emotional-regulation processes relevant to pain, fear, and anxiety. Nevertheless, the magnitude and clinical importance of these effects should be interpreted in the context of the observed effect sizes, absolute between-group differences, and the methodological limitations of the present study. Therefore, the current findings support further evaluation of VR rather than its unqualified routine implementation in pediatric venipuncture care.

### Limitations and Strengths of the Study

The strengths of the study are the random assignment of patients to groups, the presence of a control group, the application of venipuncture in line with the literature, the presence of a suitable environment that prevents patients from being affected by each other during the application, and the allocation of time for patients to perform different applications after the study is completed. The lack of opportunity for patients to perform the application before the procedure has been determined as a limitation of the study as it may negatively affect the patient.

Regarding external validity, the present findings apply most directly to school-age children (6–12 years) undergoing a single, relatively brief venipuncture procedure in an inpatient setting at a private hospital. Generalization to younger children, adolescents, outpatient or emergency settings, children with chronic conditions requiring repeated venipunctures, or children with pre-existing needle phobia or neurodevelopmental conditions should be made cautiously, as these populations were not represented in the present sample and may respond differently to VR-based distraction. Multicenter studies including public hospitals, outpatient clinics, and more diverse pediatric populations are needed to establish the generalizability of these findings.

In addition to the inability to familiarize children with the VR device beforehand, several further limitations should be acknowledged. First, the sample size was relatively small (n = 30 per group) and drawn from a single private hospital, which limits statistical power and the generalizability of the findings to other settings, including public hospitals and outpatient clinics. Second, as noted above, full blinding of participants and the treating researcher was not feasible given the visible nature of the intervention, and outcome assessors were not blinded to treatment allocation, which may have introduced expectancy or observer effects despite the blinded cross-checking of ratings between assessors. Third, potentially important confounding variables such as children’s prior venipuncture experience, parental anxiety levels, prior hospitalizations, use of analgesics or sedatives, and the technical difficulty of the venipuncture procedure were not systematically measured or statistically controlled for, and may have influenced the observed differences in pain, fear, and anxiety. Fourth, the study lacked an active distraction comparator (e.g., cartoons, bubble blowing, or a tablet-based video), so it cannot be determined whether VR is superior to simpler, lower-cost distraction strategies rather than merely superior to no distraction. Fifth, the trial was registered retrospectively, which does not meet the standard for prospective clinical trial registration. Finally, outcomes were assessed only immediately before and after the procedure, and no longer-term follow-up data on pain memory, needle-related fear, or healthcare avoidance were collected.

## 5. Conclusions

In this single-center randomized controlled trial, school-age children who used virtual reality glasses during venipuncture reported lower pain, fear, and anxiety than children in the control group, with effect sizes in the small-to-medium range; these findings are consistent with, and add to, the existing literature. Given the relatively small sample, single-center design, incomplete blinding, and absence of an active distraction comparator, these results should be interpreted as preliminary support for VR as a non-pharmacological adjunct rather than as definitive evidence. We therefore recommend a cautious approach: nurses and pediatric care teams may consider VR as one option among non-pharmacological strategies for procedural pain, fear, and anxiety management, but broader implementation in inpatient care should await confirmation in larger, multicenter trials involving more diverse pediatric populations, outpatient and emergency settings, and comparison against active distraction techniques.

As the participants were children under 18 years of age, written informed consent was obtained from their parents or legal guardians before enrollment. In addition, verbal assent appropriate to the children’s age and developmental level was obtained from each child. The children and their parents or legal guardians were provided with detailed information regarding the purpose, scope, duration, and procedures of the study, the expectations of participants, and the intended use of the collected data through an Informed Consent Form prepared in accordance with the Declaration of Helsinki. They were given sufficient opportunity to ask questions and receive detailed explanations before deciding whether to participate. Separate written consent was also obtained from the parents or legal guardians for the use of participants’ images in scientific publications.

Prior to data collection, written permission to use the study instruments was obtained via e-mail from the original developers of the measurement scales.

Participants and their parents or legal guardians were assured that all personal information would remain confidential, would be accessible only to the research team, would not be disclosed to third parties, and would be used solely for the purposes of this research. The principles of confidentiality and privacy were strictly maintained throughout the study.

Upon completion of the study, children in the control group were also offered the opportunity to experience the virtual reality intervention to ensure fairness and equal access to the educational intervention in accordance with ethical principles.

## Figures and Tables

**Table 1:** Characteristics of children and parents (n = 60).

Child Characteristics	VR Group	Control Group		
	(n = 30)	(n = 30)		
	n (%)	n (%)	X^2^	*p*
Gender				
Boy	9 (%30)	11 (%36.7)	0.300	0.392
Girl	21 (%70)	19 (%63.3)		
	M ± SD	M ± SD		
Age (year)	8.96 ± 2.09	8.83 ± 2.06		0.805
Parent characteristics				
Mother age (year)	35.10 ± 5.68	34.66 ± 5.54		0.766
Father age (year)	37.233 ± 5.56	35.73 ± 5.73		0.308
	n (%)	n (%)		
Mother’s level of education				
High school	20 (%66.7)	13 (%43.3)	3.300	0.059
University	10 (%33.3)	17 (%56.7)		
Father’s level of education				
High school	18 (%60)	13 (%43.3)	1.669	0.151
University	12 (%40)	17 (%56.7)		
Level of economic				
Low	3 (%10)	0 (%0)	5.231	0.073
Middle	21(%70)	18 (%60)		
High	6 (%20)	12 (%40)		
Type of family				
Nuclear	26 (%86.7)	28 (%93.3)	0.741	0.335
Extended	4 (%13.3)	2 (%6.7)		

X^2^: chi-square test.

**Table 2:** Comparison of pain scores before and after the venipuncture according to group (n = 60).

Procedural Pain Scores by WBS	VR Group	Control Group		
	(n = 30)	(n = 30)		
	M ± SD	M ± SD	t ^a^	*p*
Before	1.15 ± 0.43	1.17 ± 0.54	0.173	0.863
After	0.68 ± 0.51	1.06 ± 0.62	2.559	**0.013**
t ^b^	5.193	1.977		
*p*	**0.000**	0.058		

^a^ Independent Groups *t*-test; ^b^ Dependent Groups *t*-test.

**Table 3:** Comparison of fear scores before and after the venipuncture according to group (n = 60).

Procedural Fear Scores by CFS	VR Group	Control Group		
	(n = 30)	(n = 30)		
	M ± SD	M ± SD	t ^a^	*p*
Before	1.23 ± 0.56	1.20 ± 0.57	−0.227	0.822
After	0.83 ± 0.50	1.12 ± 0.53	2.157	**0.035**
t ^b^	4.397	1.270		
*p*	**0.000**	0.214		

^a^ Independent Groups *t*-test; ^b^ Dependent Groups *t*-test.

**Table 4:** Comparison of anxiety scores before and after the venipuncture according to group (n = 60).

Procedural Anxiety Scores by STAI-CH	VR Group	Control Group		
	(n = 30)	(n = 30)		
	M ± SD	M ± SD	t ^a^	*p*
Before	53.23 ± 4.32	53.96 ± 4.00	0.682	0.498
After	49.66 ± 7.29	52.86 ± 4.65	2.025	**0.048**
t ^b^	3.257	1.835		
*p*	**0.003**	0.077		

^a^ Independent Groups *t*-test; ^b^ Dependent Groups *t*-test.

## Data Availability

The data presented in this study are available from the corresponding author upon reasonable request. The data are not publicly available due to privacy and ethical restrictions involving pediatric participants.
